# Clinical Outcomes and Safety Profile of a Dextranomer–Hyaluronic Acid Hybrid Filler: A Case Series Analysis

**DOI:** 10.1111/jocd.16653

**Published:** 2024-11-06

**Authors:** Nazaret Ruiz, Roberto Miranda Lopez, Ruben Marques, Silvia Fontenete

**Affiliations:** ^1^ Private Clinic Gijón Spain; ^2^ Hospital Cruz Roja Gijón Spain; ^3^ Medical Department BioScience GmbH Madrid Spain; ^4^ Institute of Biomedicine (IBIOMED) University of León León Spain

**Keywords:** cheek augmentation, contouring, dermal filler, hyaluronic acid, mandible, volume enhancement

## Abstract

**Background:**

The demand for aesthetic treatments targeting the middle and lower face is on the rise, especially because of changes in appearance associated with aging.

**Aim:**

This study aimed to assess the use of a hybrid filler for sculpting and contouring of the chin, jaw, and malar region.

**Methods and Materials:**

A retrospective analysis was performed on patients who underwent jaw and chin contouring and cheek augmentation using a hybrid filler (hyaluronic acid and dextranomer). The evaluation focused on the naturalness of appearance, enhancement in volume, and the durability of the results, employing a 5‐point scale. Both patient satisfaction and physician evaluations were measured using the Likert scale and the Global Aesthetic Improvement Scale (GAIS), respectively. Follow‐up with patients extended up to 6 months after treatment, during which any treatment‐related adverse events (AEs) were meticulously recorded and analyzed.

**Results:**

Nineteen patients participated in the study, receiving an average injection volume of 2.4 ± 0.9 mL to attain the desired outcomes. The evaluation of natural appearance, volumizing effects, and durability at the analyzed time point consistently scored above 4. All 19 patients' aesthetic improvement was evaluated as “very much improved” and “much improved”, at the GAIS score. All patients report improvement in their appearance, with 89.5% rating it as “very much improved” or “much improved” on the Likert scale. Only expected AEs such as mild pain and lower swelling were registered.

**Conclusion:**

The hybrid filler proved effective and safe for facial contouring, with significant patient satisfaction and minimal adverse effects.

## 1 Introduction

Facial aging involves changes in both hard and soft tissues, characterized by a gradual resorption of the facial bones. This includes a backward movement of the maxilla and a downward and outward shift of the lateral and inferior orbital rims, resulting in an enlarged orbital opening. As part of the aging process, the lower jaw undergoes a kind of shrinkage both vertically and horizontally, because of the resorption at the gonial angle, chin symphysis, and the body and ramus of the jaw [1]. During the 20th century, facial rejuvenation emerged as a popular trend in beauty mainly promoted by noninvasive procedures [2].

For patients looking to avoid surgery, there is a growing demand for hyaluronic acid (HA) fillers. Moreover, surgeons have shifted toward favoring minimally invasive techniques over more invasive approaches [3]. Additionally, dermal fillers present a compelling treatment choice for addressing facial aging, offering high patient satisfaction, enduring results, and minimal risk of side effects [4]. Techniques and physiological principles tailored to these products have evolved, making it possible to achieve results that rival those of surgical interventions [5]. However, employing specific HA fillers tailored to each anatomical layer of the face is necessary to prevent unnatural appearances, particularly to avoid the filler becoming noticeable during facial movements [6].

Research in field indicates that combining HA with dextranomer is a highly effective strategy for facial rejuvenation [7]. This effectiveness is attributed to the synergistic effects of HA's hydrating and volumizing properties and dextranomer's ability to promote neocollagenesis and provide a lifting effect [8, 9]. Yet, its effectiveness as a dermal filler for enhancing the malar and mandibular areas remains unexplored.

A commercially available hybrid filler, blending hyaluronic acid and dextranomer (produced by BioScience GmbH, Germany), stands out because of its distinctive composition and is designed for facial sculpting and contouring. This case series aimed to assess the effectiveness and safety of this hybrid filler for nonsurgical contouring and augmentation of the jaw, chin, and malar area.

## 2 Methods

### 2.1 Subject Selection and Study Design

This retrospective and observational case series includes patients seeking nonsurgical lower jaw, chin sculpting, and cheek augmentation. Procedures were performed by the senior author from January 2022 to January 2023 at one single clinic. The participants were treated as part of routine clinical practice at the aesthetic clinics, with data collected from preexisting records in the database.

Male or female subjects aged 18 years or older treated with hybrid filler were included in this study. Exclusion criteria encompassed patients with known allergies to filler ingredients, those with compromised immune systems, coagulation disorders, active infections, inflammation, or acute or chronic skin diseases. Subjects with a history of facial tissue augmentation or aesthetic facial surgery within the past 6 months, pregnant or breastfeeding individuals were also excluded from treatment. Every procedure was performed at least 7 days after other aesthetic treatments.

The study was carried out by the principles of the Declaration of Helsinki and the International Conference on Harmonization Good Clinical Practice guidelines, and all patients received information about the product and procedure and signed an informed consent form for the procedure and for their anonymized clinical data to be used for training and scientific purposes.

### 2.2 Injection Filler

For this procedure, a hybrid filler composed of crosslinked HA (14 mg) and dextranomer (50 mg) (BioScience GmbH) was used. The crosslinked HA portion is obtained with 1,4‐butandioldiglycidyl ether. This filler is indicated for intracutaneous and subcutaneous injection and is characterized by the higher G′.

### 2.3 Injection Technique

Each treatment session, conducted in an outpatient setting, lasted between 10 and 30 min. Written consent was secured from each patient before the procedure. Patients underwent one session of hybrid filler treatment and returned after 15 days for possible touch‐up injections if necessary. Before injection, makeup was removed from the overlying skin, which was then sterilized with an aqueous chlorhexidine solution. To minimize discomfort, a local anesthetic was applied at the cannula entry points, using a 2% lidocaine solution. No antibiotics, corticosteroids, painkillers, or other drugs were prescribed to the patients at the end of the procedure. After the injections, Vit K cream was applied to the treated area, followed by the placement of a cold patch for 10 min to minimize postinjection redness and swelling.

#### 2.3.1 Chin Reshaping

**FIGURE 1:**
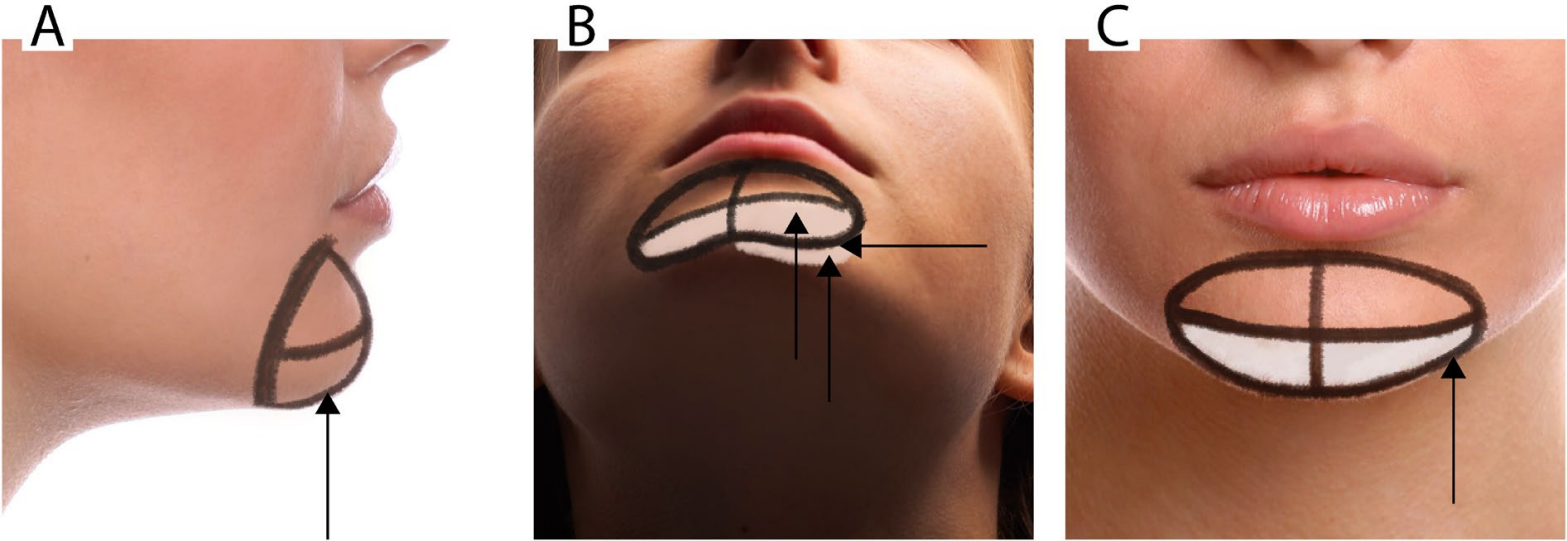
Images represent marking areas for the chin reshaping and filler injection sites. The targeted areas for filler in the chin and mental region are outlined in white. Arrows indicate the locations of the injections.

We began by marking the chin and defining its anatomical boundaries (midline and lateral limit along an imaginary vertical line descending from the corner of the mouth) and its ideal dimensions (Figure 1).

For chin reshaping, three needle entry points were established to facilitate corrections. The hybrid filler was injected into the supraperiosteal layer at the paragonion using a 25G sharp hypodermic needle and the bolus technique, aiming to enhance bone volume near the mandible's lower border and define the chin's width (Figure 1A). Another injection was administered supraperiosteally at the pogonion, 15–20 mm above the mandibular lower edge, employing the bolus method to increase bone volume for anterior chin projection (Figure 1B). Finally, using a fanning technique, the hybrid filler was introduced into the subcutaneous layer in a rhombic area outlined by the pogonion, menton, and paragonion on both sides if necessary (Figure 1C). This was done through a blunt‐tipped 25G, 3 cm cannula, entering at the initial paragonion site, aiming to augment soft tissue volume and ensure a smooth chin contour.

#### 2.3.2 Jawline Contouring

**FIGURE 2:**
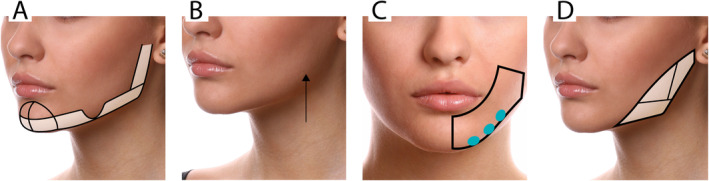
Representation of filler injection along the mandibular line. (A) The designated filling areas on the chin, lateral mental region, and along the mandibular line are highlighted in white, excluding the jowl fat area. (B) Image represents the entry point located at the mandibular angle. (C) Lower third of the face, showing three specific sites for filler injections in the supraperiosteal plane in the inferolateral mental region. (D) Injection sites along the mandibular line, including the ramus of the mandible and mandibular angle.

We marked the anatomical boundaries of the mandible, the gonial angle, and the horizontal rami (Figure 2). We also delineated the area corresponding to the jowl, which we will avoid injecting, and the pre‐jowl mandibular area (Figure 2A).

We will begin by filling the most medial part of the mandible at the pre‐jowl area, lateral to the chin (Figure 2B–D). The lower edge of the mandible was identified as the initial site for injection, where the filler was administered to create a straight jawline and correct the concavity anterior to the jowl. For this, small supraperiosteal deposits will be made using a 25G needle (Figure 2B).

At the gonial angle (Figure 2C), a perpendicular injection with a 25G needle targeted the periosteum, particularly at the angle's most lateral part, to straighten the mandible. This was done either in the posterior area to lengthen the mandible or in the most lateral part to increase lateral projection and widen the area (masculinization). Using a cannula, retrograde fillings in the subcutaneous layers were conducted longitudinally along the jawline to treat the area adequately. Injections were carefully avoided between the masseteric cutaneous ligament and the mandibular septum to prevent exacerbating the posterior jawline. To reduce the risk of intravascular injection, care was taken to identify facial arteries, ensuring precise placement of the filler.

#### 2.3.3 Malar Region: Cheek Augmentation

**FIGURE 3:**
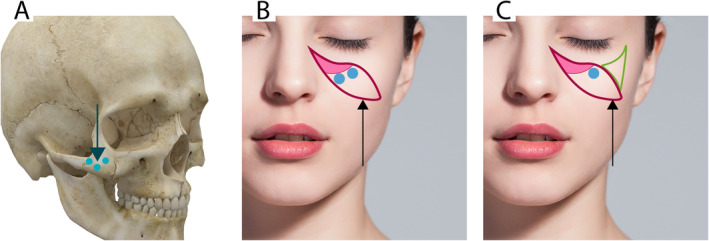
Filler injection techniques in the zygomatic and malar regions. (A) Bolus injection sites in the zygomatic area. (B) Filler injection targeting the zygomatic process. (C) Filler application along the zygomatic arch.

The procedure commenced with interventions on the lateral cheek (zygomatic area), where 2–3 bolus injections of 0.1–0.2 cc were administered using a 25G needle (Figure 3A). Following this, the medial cheek area was addressed. For the sculpting of the medial malar region, a mixed injection technique was adopted (Figure 3B,C). To augment the volume of the medial malar pouch, a 25 or 22G cannula was inserted at an entry point located 0.5 cm lateral to the nasogenian groove, oriented vertically and parallel to the skin. A bolus of 0.2–0.5 cc of hybrid filler was delivered to the targeted medial malar fat pad, ensuring placement below the orbital rim.

Injections in the more lateral malar region and at the zygomatic–malar junction were performed using a 25G needle positioned in the supraperiosteal layer. It was crucial to carefully consider the anatomical landmarks of the region to prevent damage to the infraorbital neurovascular pedicle. Following the injection, the treated areas were gently massaged to facilitate the even distribution of the filler and to achieve desired facial contouring. The procedure was repeated as necessary, with total filler volumes ranging from 0.2 to 1.2 mL per patient, to attain the intended volumetric enhancement.

### 2.4 Assessments

All assessments were conducted by the same plastic surgeon who performed the procedures. Follow‐up visits were scheduled at 48 h, 2 weeks, and 1, 3, and 6 months following the initial treatment.

Syringe force was evaluated on a scale from 1 (minimum force) to 5 (maximum force). Natural results, volumizing effect, and durability were evaluated by the investigator on a 5‐point scale, where 1 is the minimum score and 5 is the maximum score.

Global Aesthetic Improvement Scale (GAIS) was used to analyze the aesthetic improvement in appearance (4 weeks) compared with pretreatment. The GAIS is a 5‐level scale: very much improved (1) = optimal cosmetic result; much improved (2) = market improvement in appearance but not completely optimal, and a touch‐up would slightly improve the result; improved (3) = obvious improvement in appearance from the initial condition, but a touch‐up or re‐treatment is indicated; no change (4) = the appearance is essentially the same as the original condition; and worse (5) = the appearance is worse than the original condition.

Subject satisfaction was evaluated at 4 weeks using the Likert scale, that is, at 1 month after treatment. The Likert scale is a scale that is rated as follows: A = very dissatisfied, B = dissatisfied, C = slightly satisfied, D = satisfied, and E = very satisfied.

Adverse events (AEs) were categorized as unexpected and undesirable events or common and expected events that developed after the treatment. They were evaluated from patient diaries self‐recorded for all the study and patient interviews at each follow‐up visit. Additionally, two common and expected events, pain and swelling, were evaluated up to 48 h after injection. Patients rated injection pain on a scale from 1 to 5 (with 1 indicating the least pain and 5 indicating the most pain). The scale was defined as follows: 0 indicated the absence of pain; 1 represented mild pain; 2 described moderate pain; 3 corresponded to severe pain; 4 represented very severe pain; and 5 indicated worse pain possible.

The swelling effect at 48 h postinjection was assessed by the physician. Swelling was assessed using a 5‐point scale to categorize its severity 48 h posttreatment. The scale was defined as follows: 1 indicated no swelling; 2 represented subjective swelling that was not noticeable to others; 3 described mild to moderate swelling that resolved within 24 h; 4 corresponded to moderate swelling that did not double the immediate posttreatment volume and persisted for more than 24 h; and 5 indicated severe swelling that doubled the posttreatment volume and lasted for more than 24 h.

### 2.5 Data Analysis

Descriptive statistical analyses were performed to summarize the data in this study. Measures of central tendency and dispersion, including mean and standard deviation (SD), were calculated to describe continuous variables. For categorical variables, percentages were used to represent the distribution of scores across different groups. All analyses were conducted using standard statistical software (GraphPad Prism 10), with results presented as mean ± SD for continuous data and as percentages for categorical data, ensuring a clear representation of distribution patterns within the sample.

## 3 Results

### 3.1 Patient Characteristics and Demographics

**FIGURE 4:**
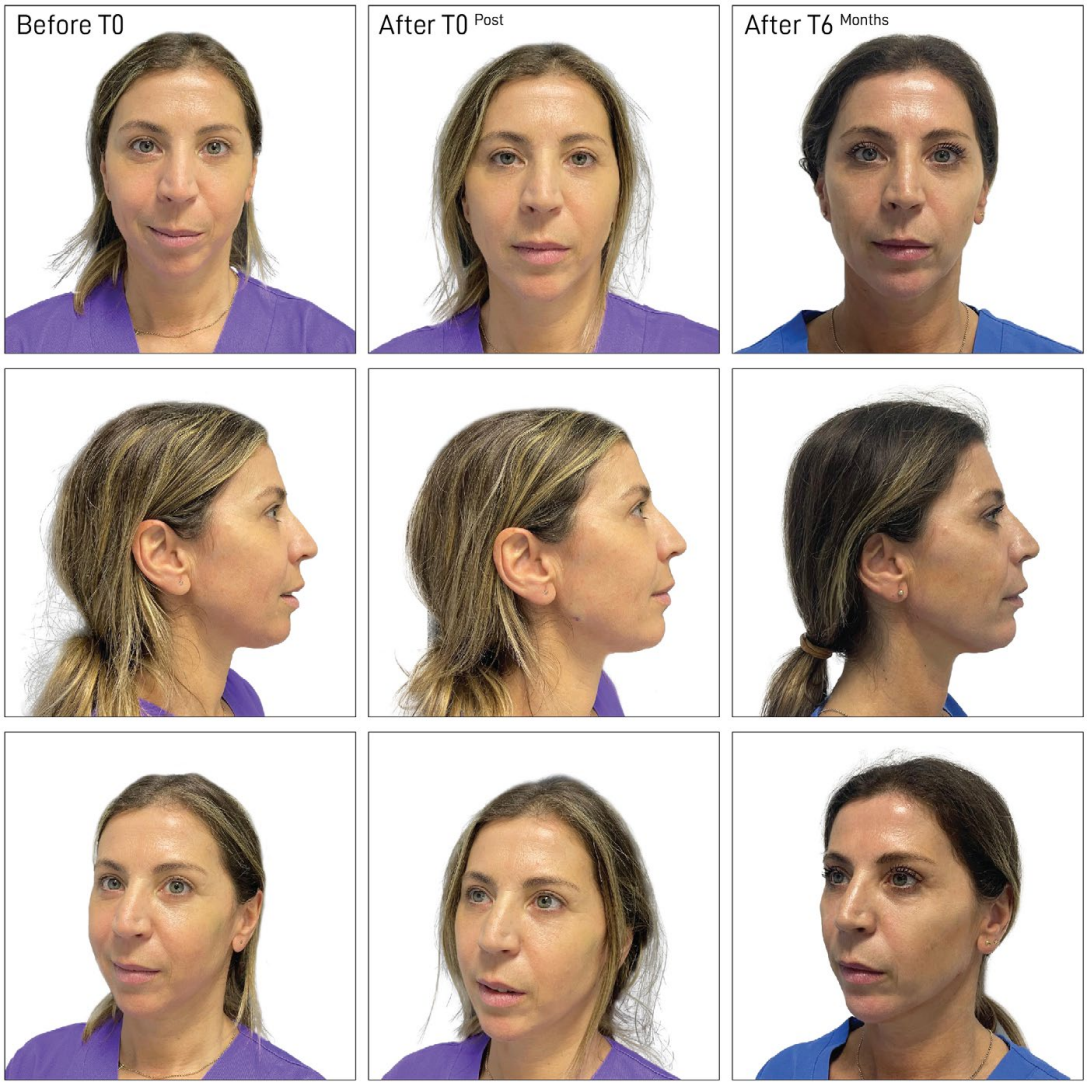
Case 1: The upper images show the patient's frontal view, the middle images display the lateral view, and the lower images present the angled view. The left column represents images taken before treatment (before T0), the middle column shows images immediately after treatment (after T0), and the right column displays images taken 6 months posttreatment (after T6).

**FIGURE 5:**
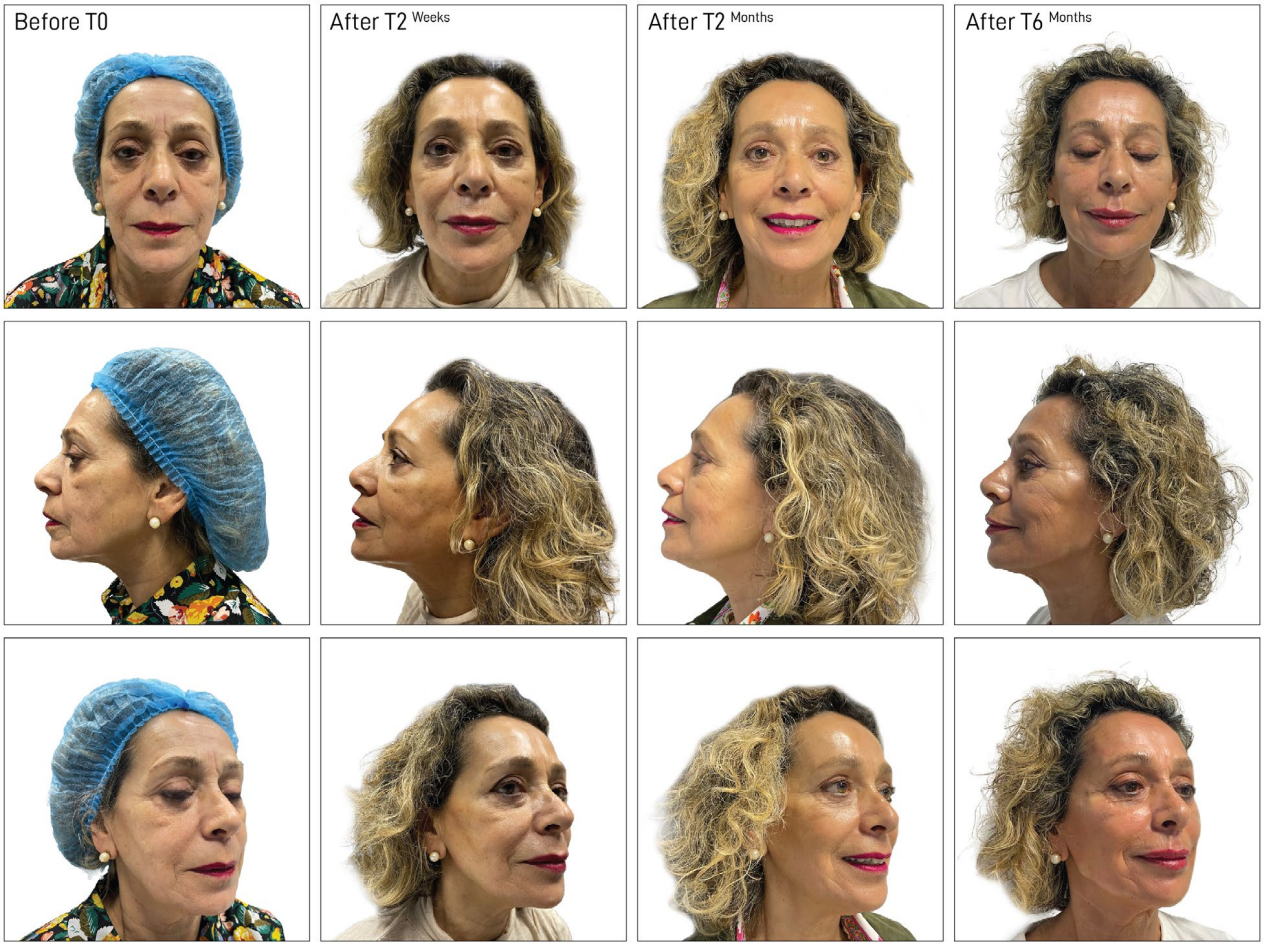
Case 2: The upper images display the patient's frontal view, the middle images show the lateral view, and the lower images present the angled view. The first (left) column represents images taken before treatment (T0 before), the second column shows images taken 2 weeks posttreatment (after T2), the third column displays images taken 3 months posttreatment (after T3). The final (right) column presents images taken 6 months posttreatment (after T6).

Nineteen patients seeking nonsurgical reshaping of the jawline or/and cheek augmentation (malar reshaping) were examined and treated. Some representative cases are shown in Figures 4 and 5; Figures S1 and S2. Most of the subjects were female (16/19, 84.2%), with a mean age of 53 years (Table 1). The minimum follow‐up was 1 month, and the maximum up to 6 months. Not all patients appeared for every follow‐up at 3 and 6 months. A minimum of one and up to three vials of filler was injected to achieve the desired results. The demographics and baseline characteristics of the study population are summarized in Table 1.

**TABLE 1:** Demographics and baseline characteristics of the study population.

Characteristics	Total (*n* = 19)
Age, years	
Minimum Maximum	38 63
Gender, *n* (%)	
Male	3 (15.8)
Female	16 (84.2)
Race, *n* (%)	
Caucasian	19 (100.0)

### 3.2 Syringe Force

Syringe force was analyzed by the investigator in each procedure on a scale of 1 to 5 (from minimum to maximum) with an average of 4.0 ± 0.0.

### 3.3 Efficacy

**FIGURE 6:**
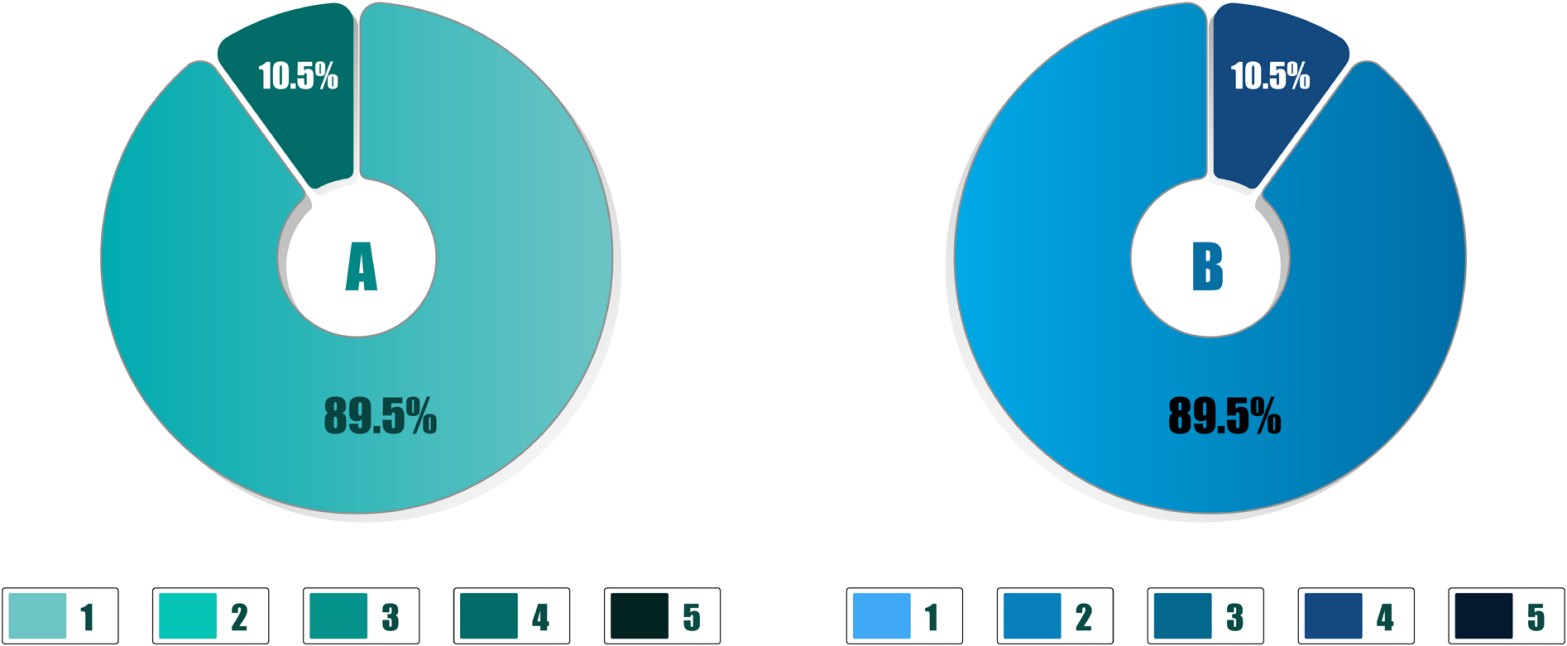
Evaluation of aesthetic results using GAIS (A) and Likert scale (B).

The GAIS was used by the investigator to evaluate the aesthetic improvement of the treatment. All 19 patients had a 1 or 2 score (“very much improved” and “much improved”) at 4 weeks. Most patients had a 1 as a rated GAIS score (17/19, 89.5%) with an average of 1.1 ± 0.3 (Figure 6A).

Patients evaluated the aesthetic enhancements at 4 weeks using the Likert scale. Every participant (100%) reported improvements, categorizing them as either “very much improved” or “much improved” during the study period. A significant majority of the patients (17 out of 19, or 89.5%) described their improvement as “very much improved” (Figure 6B).

**FIGURE 7:**
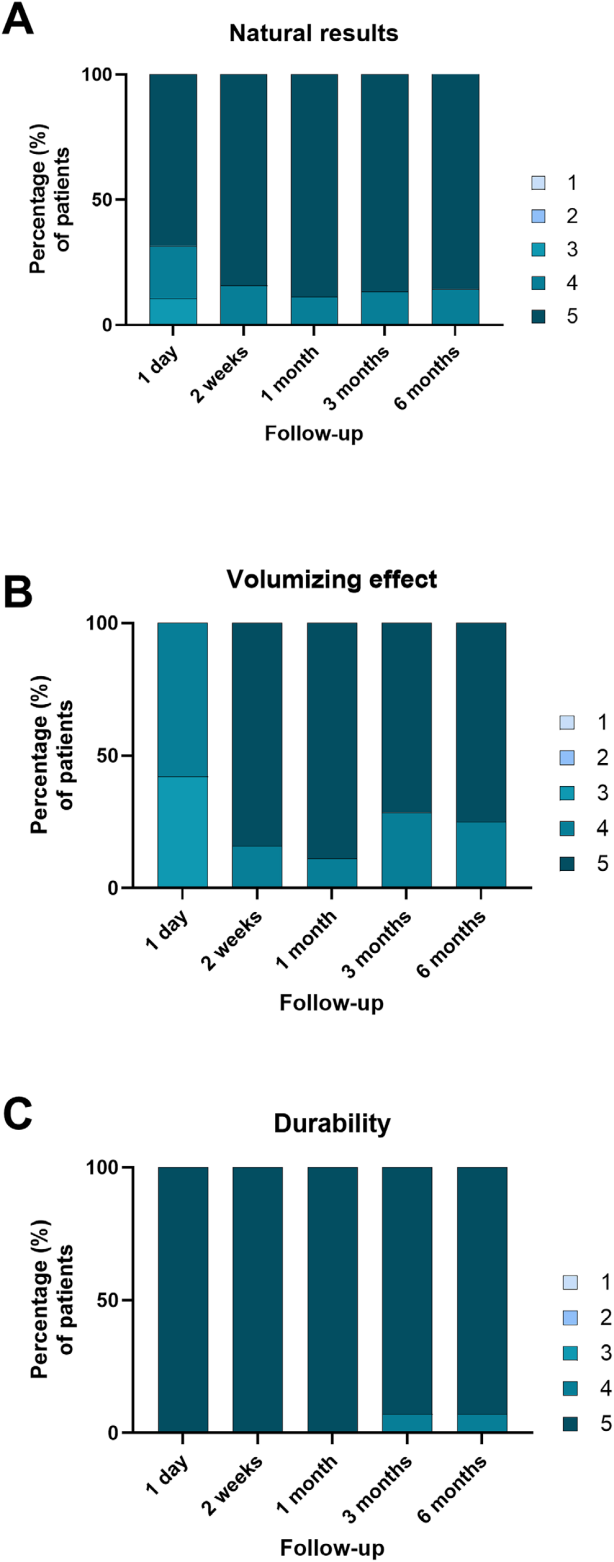
Evaluation of aesthetic outcomes was conducted using a 5‐point scale for natural appearance (A), volumizing effect (B), and durability (C). The graph illustrates the percentage of patients across the different scoring levels.

Natural effect and volumizing results were evaluated by the investigator using a 1–5 score scale. Results are described in Figure 7A,B. The mean score for natural results is 4.8 ± 0.3 and for volumizing results is 4.6 ± 0.4.

### 3.4 Durability

The durability of the effect was evaluated using a 1–5 score scale by the investigator during all the follow‐up and is represented in Figure 7C. The mean score for durability is 4.9 ± 0.2.

### 3.5 Safety

The most observed expected AEs following the injections were swelling and pain, affecting 15.8% of the participants (3 out of 19 patients). Additionally, one patient experienced hematoma in the malar region, which resolved spontaneously 7 days postinjection without requiring any intervention. On a scale of up to 5, patients reported an average pain (procedure) score of 3.0 ± 0.2, suggesting a moderate level of discomfort. The physician assessed the swelling following the procedure as low, assigning a score of 2.1 ± 0.2 on a scale of up to 5.

All patients were revisited 2 weeks after the initial procedure and exhibited minimal inflammation. No additional AEs were noted by the treating physician during follow‐up visits, nor were any reported by the patients. There were no instances of severe AEs. Throughout the study, patients did not report experiencing tenderness, infection, or the Tyndall effect.

## 4 Discussion

As the face ages, it is prone to losing volume, which can lead to chin ptosis, diminished definition, and the accumulation of excess skin [10]. Sculpting the jawline and chin, along with achieving high cheekbones and a full malar area, has gained considerable popularity for its role in enhancing facial attractiveness, as a well‐defined demarcation significantly enhances attractiveness and is increasingly recognized as an essential component to complete facial aesthetic rejuvenation [11, 12, 13, 14]. Although autologous fat transfer offers a viable solution for many, its major limitation lies in the unpredictable duration of its effects, possibly necessitating a follow‐up procedure in the future [4]. Dermal fillers offer a nonsurgical approach to facial aesthetic rejuvenation. Research in field has demonstrated that injectable fillers can effectively and safely enhance the jawline, chin, and cheeks [15, 16, 17]. In this case series study, we showcase the outcomes of employing a hybrid filler for sculpting the jawline, chin, and cheeks.

In our research, we noted that all participants showed significant improvement, either “very much improved” or “much improved” according to the GAIS and Likert scale, following the use of a hybrid filler for enhancing the jawline, chin, and malar area. The effectiveness of this hybrid HA filler, which includes dextranomer components, has been similarly documented in other studies within the literature that used fillers with similar composition. Shin et al.'s study evaluates the efficacy of subcutaneous injection of a dextran filler in treating nasolabial folds for 24 weeks. The authors observed there were significant improvements (*p* < 0.0001) in the Wrinkle Severity Rating Scale scores compared with those at baseline with a mean decrease of 1.50 ± 0.51 at 24 weeks [7]. The effectiveness of combining HA and dextranomer can be attributed to the mechanism induced by dextranomer. Eppley and colleagues have explained that the positive surface charges on dextran beads attract macrophages. These macrophages then secrete TGF‐beta and interleukins, which in turn stimulate fibroblasts to produce collagen fibers [8].

Outcomes of HA fillers are also attainable with an in‐depth knowledge of the relevant anatomy and meticulous patient selection. Recognizing gender distinctions is crucial, especially given the jaw's significant role in determining gender perception when assessed separately. Typically, male faces exhibit a square shape with characteristics such as a wider mandible, a more substantial masseter muscle, and a broader, more pronounced chin. Conversely, female faces are generally more triangular, showcasing a slender chin and jawline. Additionally, there is a notable variation in the preferred sites for filler injections between genders [18]. Another aspect that is necessary to take into consideration is understanding patients' aims and motivation and performing an individualized treatment approach instead of a “whole‐face” approach [19].

Regarding the safety profile, the AEs recorded were expected, limited to minor, transient reactions at the injection site. We noted that mild pain (pain score of 3.0 ± 0.2) and slight swelling (score of 2.1 ± 0.2) were recorded as expected AEs, with no additional AEs observed throughout the patient follow‐up period. There were no reports of serious AEs, including allergic reactions, infections, granulomas, skin necrosis, or the Tyndall effect. Similarly, Shin et al.'s study demonstrated the safety of the dextran filler, where only one mild treatment‐related AE was reported over a 24‐week follow‐up. Furthermore, the application of the dextranomer component in various medical fields has consistently shown a safe profile [20, 21, 22]. Moreover, unlike certain components found in other hybrid HA fillers, dextranomer microspheres are known to be completely reabsorbed [23].

This study's limitations stem from its small sample size and short duration; thus, further research is needed to assess long‐term safety and effectiveness in a prospective randomized controlled trial. One limitation of this study is that all assessments were conducted by the same plastic surgeon who performed the procedures, leading to a lack of blinding in the study design. The authors acknowledge the absence of measurable outcomes because of the current unavailability of appropriate clinical technologies, such as 3D cameras, and plan to address this limitation in future studies. However, treatment results were appraised using two aesthetic improvement metrics: the Likert scale and the GAIS.

Despite the study's limitations, including a small patient cohort and a duration of only 6 months, it successfully showed favorable results using a hybrid filler for facial treatments.

## 5 Conclusions

This case series study shows that a hybrid filler made of HA and dextranomer is safe and effective for enhancing the malar and mandibular facial regions. The observed AEs were limited to short‐lived reactions at the injection sites, and the Tyndall effect was not observed.

## Author Contributions

N.R. and R.M.L. designed the study and performed the treatment with HA fillers and the follow‐ups of the subjects. S.F. performed data analysis. S.F. and R.M. wrote the manuscript. All the authors reviewed and approved the final version of the manuscript.

## Ethics Statement

All procedures were performed according to the 1964 Helsinki Declaration and its later amendments or comparable ethical standards.

## Consent

All participants gave their written consent for participation in the study.

## Conflicts of Interest

S.F. and R.M. are employees at BioScience GmbH.

## Supporting information


**Figure S1.** Case 3: Images before treatment (before T0), immediately after treatment (after T0), and 1 week posttreatment (after T1).

**Figure S2.** Case 4: Upper images show the frontal view of the patient, and lower images show the lateral view. Images are presented before treatment (before T0), immediately after treatment (after T0), and 2 weeks posttreatment (after T2).

## Data Availability

The data that support the findings of this study are available from the corresponding author upon reasonable request.
