# Supplementary figures and images for: Clinical Outcomes and Safety Profile of a Dextranomer–Hyaluronic Acid Hybrid Filler: A Case Series Analysis
Source: J Cosmet Dermatol. 2024 Nov 6;24(1):e16653. doi: 10.1111/jocd.16653 (PMC11743303; doi:10.1111/jocd.16653)

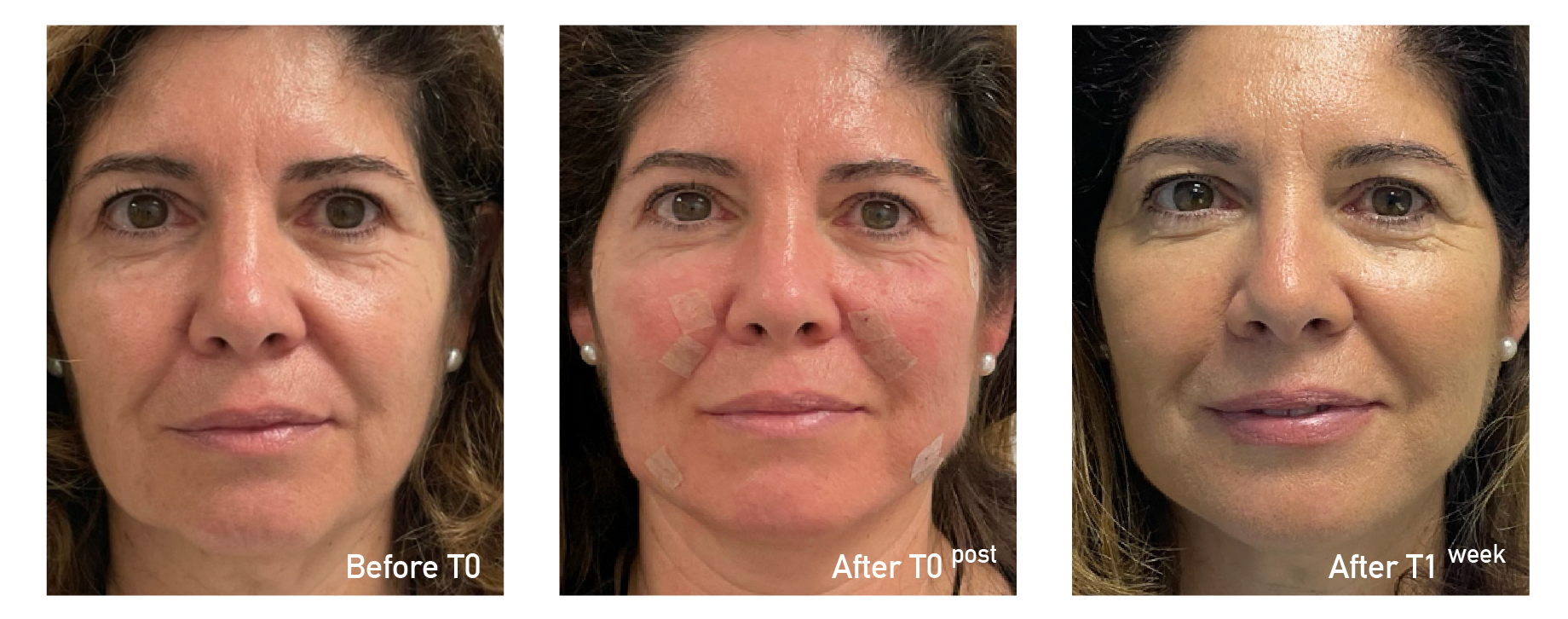

Supplement: Supplementary file 1 — Figure S1. Case 3: Images before treatment (before T0), immediately after treatment (after T0), and 1 week posttreatment (after T1). [file JOCD-24-e16653-s002.png]

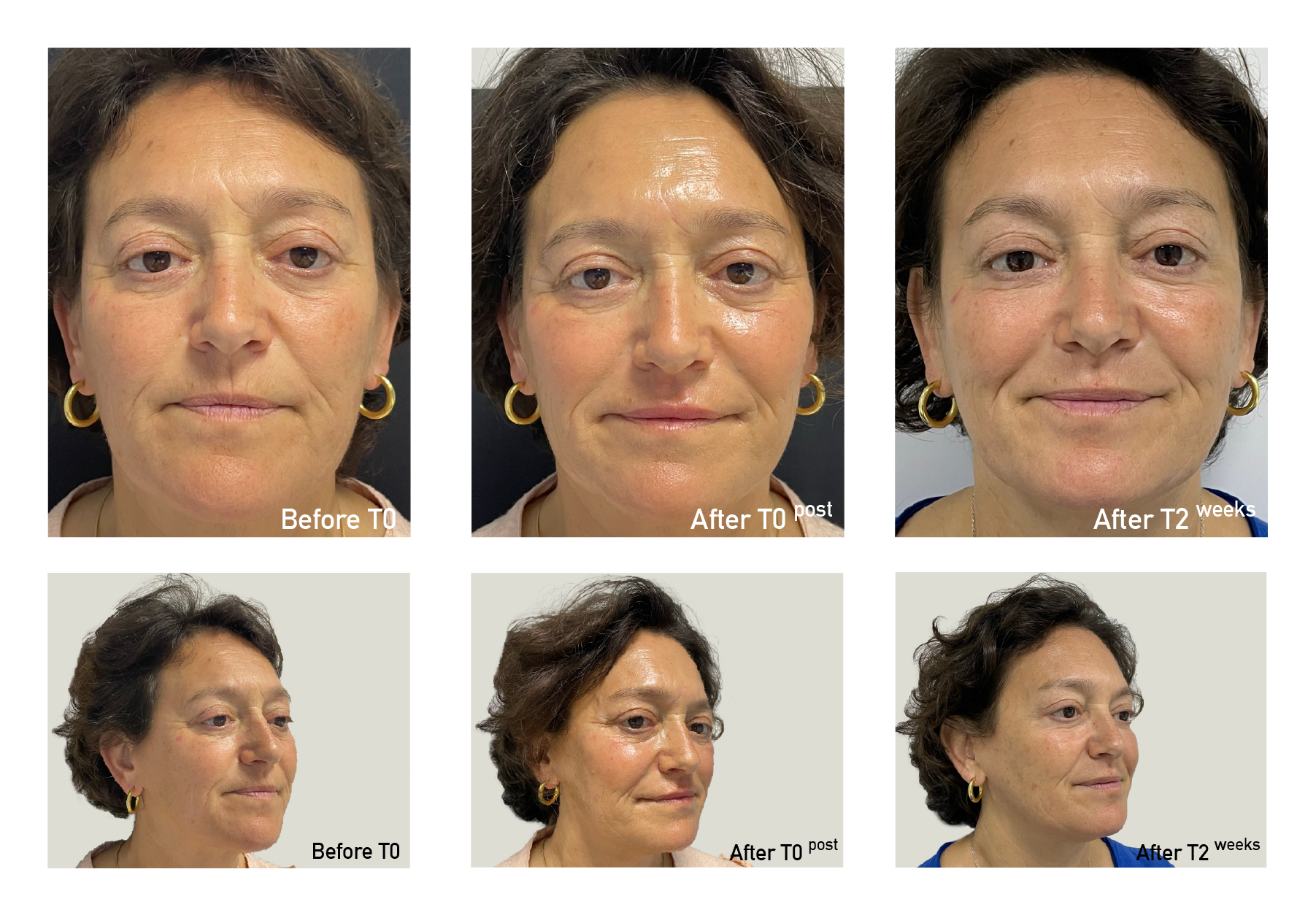

Supplement: Supplementary file 2 — Figure S2. Case 4: Upper images show the frontal view of the patient, and lower images show the lateral view. Images are presented before treatment (before T0), immediately after treatment (after T0), and 2 weeks posttreatment (after T2). [file JOCD-24-e16653-s001.png]
